# Associations between Active Trachoma and Community Intervention with Antibiotics, Facial Cleanliness, and Environmental Improvement (A,F,E)

**DOI:** 10.1371/journal.pntd.0000229

**Published:** 2008-04-30

**Authors:** Jeremiah Ngondi, Fiona Matthews, Mark Reacher, Samson Baba, Carol Brayne, Paul Emerson

**Affiliations:** 1 Department of Public Health and Primary Care, Institute of Public Health, University of Cambridge, Cambridge, United Kingdom; 2 MRC Biostatistics Unit, Institute of Public Health, Cambridge, United Kingdom; 3 Health Protection Agency, East of England Regional Epidemiology Unit, Institute of Public Health, Cambridge, United Kingdom; 4 Ministry of Health, Government of Southern Sudan, Juba, Sudan; 5 The Carter Center, Atlanta, Georgia, United States of America; University of California San Francisco, United States of America

## Abstract

**Background:**

Surgery, Antibiotics, Facial cleanliness and Environmental improvement (SAFE) are advocated by the World Health Organization (WHO) for trachoma control. However, few studies have evaluated the complete SAFE strategy, and of these, none have investigated the associations of Antibiotics, Facial cleanliness, and Environmental improvement (A,F,E) interventions and active trachoma. We aimed to investigate associations between active trachoma and A,F,E interventions in communities in Southern Sudan.

**Methods and Findings:**

Surveys were undertaken in four districts after 3 years of implementation of the SAFE strategy. Children aged 1–9 years were examined for trachoma and uptake of SAFE assessed through interviews and observations. Using ordinal logistic regression, associations between signs of active trachoma and A,F,E interventions were explored. Trachomatous inflammation-intense (TI) was considered more severe than trachomatous inflammation-follicular (TF). A total of 1,712 children from 25 clusters (villages) were included in the analysis. Overall uptake of A,F,E interventions was: 53.0% of the eligible children had received at least one treatment with azithromycin; 62.4% children had a clean face on examination; 72.5% households reported washing faces of children two or more times a day; 73.1% households had received health education; 44.4% of households had water accessible within 30 minutes; and 6.3% households had pit latrines. Adjusting for age, sex, and district baseline prevalence of active trachoma, factors independently associated with reduced odds of a more severe active trachoma sign were: receiving three treatments with azithromycin (odds ratio [OR] = 0.1; 95% confidence interval [CI] 0.0–0.4); clean face (OR = 0.3; 95% CI 0.2–0.4); washing faces of children three or more times daily (OR = 0.4; 95% CI 0.3–0.7); and presence and use of a pit latrine in the household (OR = 0.4; 95% CI 0.2–0.9).

**Conclusion:**

Analysis of associations between the A,F,E components of the SAFE strategy and active trachoma showed independent protective effects against active trachoma of mass systemic azithromycin treatment, facial cleanliness, face washing, and use of pit latrines in the household. This strongly argues for continued use of all the components of the SAFE strategy together.

## Author Summary

Trachoma is an infectious disease that is cased by a bacterium, *Chlamydia trachomatis*, and is the leading cause of preventable blindness estimated to be responsible for 3.6% of blindness globally. The World Health Organization (WHO) recommends a strategy for trachoma control known as SAFE—surgery, antibiotics, facial cleanliness, and environmental improvement. Regular evaluations of trachoma control activities are advocated for by the WHO for decision making, programme planning, and the rational use of programme resources. We undertook a survey to evaluate the effectiveness of the SAFE strategy following three years of interventions in four districts in Southern Sudan. In this paper, we aimed to find out the relationship between the antibiotics, facial cleanliness, and environmental improvement (A,F,E) and active trachoma signs. Our study revealed that prevalence of active trachoma was less in children who had received treatment with azithromycin, had clean faces, had faces washed more frequently, and used latrines compared to children who had not received these interventions. The study findings are important since they make the case for implementing the A,F,E interventions together.

## Introduction

The World Health Organization (WHO) promotes the SAFE strategy for trachoma control (Box 1) which comprises: 1) Surgery, eyelid surgery to correct in-turned eyelashes that stops pain and minimizes risk of corneal damage [1]; 2) Antibiotics, treatment for active trachoma using single-dose oral azithromycin or tetracycline eye ointment [2]; 3) Facial cleanliness, clean faces especially in children through sustained behaviour change [3]; and 4) Environmental improvement, to increase access to water and sanitation [4]. Antibiotic therapy in individuals and facial cleanliness in children, combined with environmental improvement (A,F,E components of SAFE), have been designed to treat ocular Chlamydia infection and reduce the risk of transmission of ocular Chlamydia.

There is evidence from randomised controlled trials that the individual A,F,E components of the SAFE strategy have an effect on active trachoma when applied on their own: effect of antibiotics on active trachoma at three months [2]; effect of face-washing on trachomatous inflammation-intense (TI) [3]; and effect of fly-control on active trachoma at three and six months [5],[6]. In these trials, the effects of these components have been tested individually to avoid the use of hybrid interventions that generate findings that are difficult to interpret. The challenge of disentangling the relative contribution to an effect of a combination of components has prevented trials of such hybrid trials being conducted. However, the reality of program implementation is that SAFE is a comprehensive and integrated strategy that has three control components (the A,F,E) that should be applied simultaneously.

In the biological and epidemiological context, it is reasonable to expect there to be an additive effect of the A,F,E components of the SAFE strategy. Mass azithromycin administration, plus an increase in clean faces among children, plus improved access to water, plus reduced vector populations will likely be more effective than any one component alone. Despite the need for evaluation of the combined effects of A,F,E components of SAFE being highlighted previously [7], no studies have explored the relationship between programme delivery of the combined A,F,E interventions and prevalence of active trachoma. Therefore we aimed to investigate the association between active trachoma and community intervention with A,F,E components of the SAFE strategy.

## Methods

### Study population

**Figure 1:**
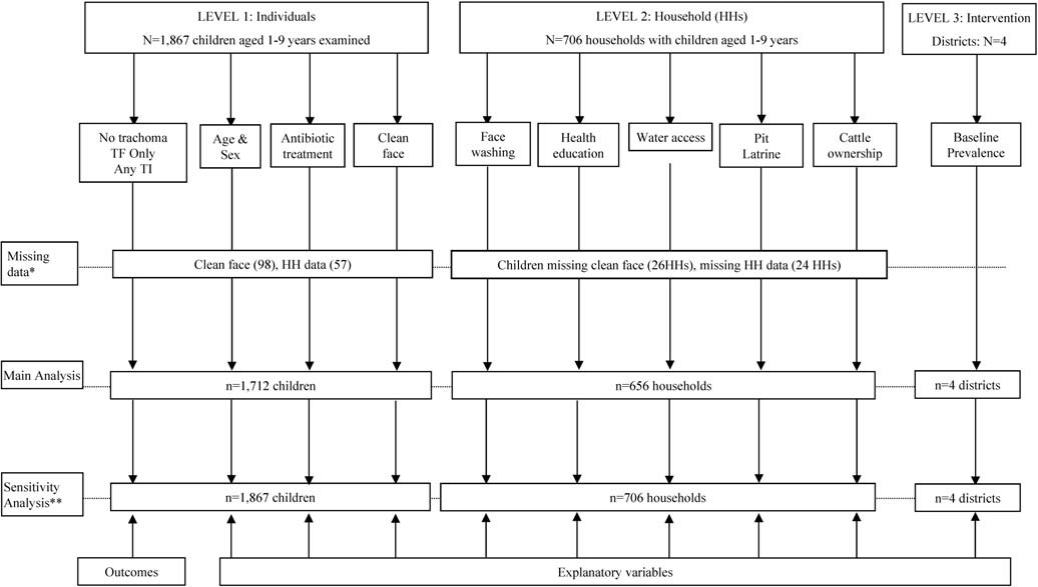
Summary of data framework for analysis of association between active trachoma and A, F, E interventions. AFE = Antibiotics, Facial cleanliness, Environmental improvement. *98 children in 26 households with missing clean face data, and 24 households (with 57 children) missing household data. **Multiple imputations of missing data.

Surveys were conducted in four districts (Kiech Kuon, Padak, Katigiri and Tali) in Southern Sudan between April to June 2005. The sample size estimation and sampling of the surveys has been described previously [8]. In brief, population based surveys were undertaken to estimate the prevalence of active trachoma signs before and following three years of implementation of the SAFE strategy. A two-stage cluster random sampling design was used to select the sample. In each district, 6 villages (clusters) were selected in stage one and 30 households selected in each cluster at the second stage. All eligible persons residing in the household were examined for trachoma and/ or interviewed. Only children aged 1–9 years, who had been examined for trachoma signs, were included in the sample (Figure 1).

### Outcomes: Severity of active trachoma signs

Eligible children were examined for trachoma signs by integrated eye care workers (IECW) using the WHO simplified grading scheme [9]. Trainee examiners had to achieve at least 80% interobserver agreement in identifying trachoma signs compared to the senior examiner to participate in the survey [10]. Clinical signs of inflammatory trachoma (TF, and TI) were graded for each eye separately. An ordinal severity score of active trachoma comprising three categories was then assigned to all eligible participants based on the worst affected eye: where ‘1’ was no TF, no TI; ‘2’ TF only; and ‘3’ TI with or without TF. We have previously justified the ordinal nature of inflammatory signs of trachoma [11]: 1) pathogenesis of trachoma is initially characterised by lymphoid follicles (stage TF), whereas papillary hypertrophy (stage TI) is seen with advancing severity [12]; 2) participants seen in longitudinal studies with persistent TI are more likely to progress to scarring (stage TS) than those with only TF [13]; 3) subjects with TI are more likely to provide ocular swabs positive for *Chlamydia trachomatis* than those with TF [14]; and 4) patients with TI provide ocular swabs that have a greater quantifiable load of *C. trachomatis* than those with TF [15],[16].

### Interventions: Surgery, Antibiotics, Facial cleanliness and Environmental improvement (SAFE)

Prior to implementation of the trachoma control programme, baseline surveys were conducted to establish the need for interventions and to define programme targets [17]. The SAFE strategy was implemented in accordance with standards advocated by the WHO [18]–[21] and have been described previously [8]. Non-governmental organizations (NGOs) responsible for implementing healthcare, in the absence of Ministry of Health infrastructure, undertook implementation of the SAFE strategy.

Surgery: Identification and screening of trachomatous trichiasis (TT) cases was conducted by community health workers trained in using the WHO simplified grading scheme [9]. TT surgeons were trained by an experienced ophthalmologist from the Christoffel Blindenmission (CBM). Eyelid surgery was conducted using the bilamellar tarsal rotation procedure at primary health care centers (PHCC) and at annual “surgery camps” [18]. TT surgery logs were maintained at the PHCC. Trichiasis surgery is not considered further in this study of associations between A,F,E interventions and active trachoma.

Antibiotics: Azithromycin (Zithromax, donated by Pfizer) was targeted for distribution to all villages in the four districts [19]. During the annual treatment round, teams of distributors including health care workers and trained village volunteer moved from village to village providing directly observed treatment to all eligible persons. Persons not eligible to receive Zithromax were treated with 1% tetracycline eye ointment. A tally of number of people treated with Zithromax was maintained and reported to the programme monthly. Three annual rounds were distributed in Kiech Kuon, Katigiri and Tali; however, the third round had not been distributed in Padak by the time of the evaluation survey.

Facial cleanliness: Health education and hygiene promotion on facial cleanliness was conducted by trained hygiene promoters, women-peer educators and health workers A flip chart with key messages on facial hygiene and prevention of trachoma was used routinely to deliver health education at health facilities, schools, churches and during community outreach. Within villages, trained women peer-educators coordinated facial hygiene promotion activities. Heath education was delivered at least once a month to target groups or communities. Health education and hygiene promotion activities were monitored and reported monthly.

Environmental change: Hygiene promoters and local leaders spearheaded community mobilization for construction of household pit latrines. The programme provided digging tools and technical advice on pit latrine construction. In two districts (Tali and Katigiri) fero-reinforced concrete slabs were provided free of charge to households for latrine construction. The number of latrines constructed was reported monthly. Advocacy for water provision to target populations by development NGOs involved in water activities was also conducted.

### Measurement of uptake of A,F,E Interventions

Structured interviews with mothers of children as principal household respondents and observations were used to measure the uptake of the A,F,E interventions using the following definitions.

Antibiotics: reports by care-givers of the number of times azithromycin treatment a child had received over three years.

Facial cleanliness: a clean face was defined as the absence of nasal and/or ocular discharge on examination; frequency of face washing was reported by care-givers as the number of times faces of children were washed per day; and whether heads of households had received health education on trachoma at home or elsewhere.

Environmental improvement: water access was report by people responsible for water collection as the time for a return journey to collect water; and pit latrine availability and use was ascertained by observation.

Other explanatory variables included age, sex, cattle ownership (reported and confirmed presence of cattle in the vicinity of the household), and baseline prevalence estimates of active trachoma for each study site.

### Statistical analysis

Statistical analysis was conducted using Stata 8.2 (Stata Corporation, College Station, Texas). Descriptive statistics were used to examine the sample characteristics, prevalence of active trachoma signs, prevalence of A,F,E interventions, and other explanatory variables. Differences in proportions were compared by chi-square test. To investigate the association between severity of active trachoma signs and A,F,E interventions, ordinal logistic hierarchical regression models were developed using generalized linear models (GLM) [22]. The multilevel structure of GLM allowed for non-independence of the household and district variables, enabled clustering of individual observations within households, and districts, and allowed for variability at individual, household, village and district levels. We fitted an ordinal logistic regression model to study associations between severity of active trachoma signs and A,F,E interventions [23]. This model allowed for analysis of a polytomous ordinal response on a set of predictors and computed odds ratios (OR) of having a more severe active trachoma sign compared to a less severe sign. In this model TI was considered more severe than TF, which is in turn a more severe sign of trachoma than a normal conjunctiva. This method did not assume that TF causes TI or that the relationship between the three orders (normal, TF only, and any TI) is linear. Univariate analysis was conducted for each explanatory variable. Multivariable models were then developed by stepwise regression analysis for model selection. This involved starting with a null model then proceeding in a sequential fashion of adding/deleting explanatory variables if they satisfied the entry/removal criterion which was set at 5% significance level using a log-likelihood ratio test. Age and sex were retained in all multivariable models to control for any potential confounding effects. Potential effect modification was evaluated by including interaction terms in the models. Sensitivity analyses were conducted to investigate the effect of missing data by multiple imputations [24].

### Ethical consideration

The Sudan Peoples Liberation Movement Secretariat of Health (SPLM/Health) approved the protocol and clearance to conduct the surveys was obtained from the local authorities. Verbal informed consent to participate was sought from the head of the household, household respondents and parents of children in accordance with the declaration of Helsinki. Consent for household interviews and trachoma examination was documented by interviewers and examiners on the data collection forms. Personal identifiers were removed from the data set before analyses were undertaken.

## Results

### Characteristics of the study population

A total of 25 clusters (villages) and 743 households in four districts were surveyed (in Tali district an additional cluster was selected due to insufficient number of households in one of the six primary clusters). Of the 1,867 children aged 1–9 years examined for trachoma, 1,712 (91.7%) in 656 (88.3% of 743 surveyed) households were included in the main analysis (Figure 1). Of the excluded households, 36 (4.8%) did not have eligible children, 26 (3.5%) had all eligible children (98) missing data on clean face, whereas 25 households (with 57 children) had missing data on health education and face washing frequency. The overall proportion of children aged 1–4 was 47.8%, the overall proportion of boys was 51.0%, and the mean age [standard deviation (SD)] was 4.9 years (SD = 2.5).

### Prevalence of active trachoma signs

The overall prevalence of active trachoma severity scores was: “no TF, no TI” = 64.1%, range by study district (29.7–94.8); “TF only” = 23.1%, range (4.7–41.0); and “any TI” = 12.8%, range (0.5–35.9) (Table 1).

**Table 1:** Demographic characteristics of sample population, prevalence of active trachoma signs and prevalence of explanatory variables.

Factor		District				Overall
		Kiech Kuon	Padak	Katigiri	Tali	
Children aged 1–9 years examined		574	464	415	414	1,867
Children included in analysis		485	456	381	390	1,712
*Prevalence of active trachoma signs (%)*						
Active trachoma	TF and / or TI	70.3	50.0	5.3	6.4	35.9
Trachoma severity score	No TF, no TI	29.7	50.0	94.8	93.6	64.1
	TF Only	34.4	41.0	4.7	5.9	23.1
	Any TI	35.9	9.0	0.5	0.5	12.8
*Individual characteristics* (%)						
Age group	1–4 years	47.6	50.2	42.5	50.3	47.8
	5–9 years	52.4	49.8	57.5	49.7	52.2
Sex	Male	50.5	53.7	53.8	45.6	51.0
	Female	49.5	46.3	46.2	54.4	49.0
Azithromycin treatment	None	90.5	28.3	22.6	38.5	47.0
	1 time	4.1	42.1	42.5	39.2	30.8
	2 times	3.3	29.6	29.4	20.3	20.0
	3 times	2.1	0.0	5.5	2.1	2.3
Children with clean faces (%)		44.7	41.2	95.3	77.2	62.4
Number of households include in analysis		184	172	170	180	706
*Household characteristics* (%)						
Face washing frequency per day	1 time or none	25.8	26.5	20.7	37.2	27.5
	2 times	51.8	42.8	46.5	41.3	45.8
	3 or more times	22.5	30.7	32.8	21.5	26.8
Health education	No	50.3	9.9	23.1	21.3	26.9
	Yes	49.7	90.1	76.9	78.7	73.1
Water access	Less than 30 minutes	65.2	20.6	44.9	45.9	44.4
	More than 30 minutes	34.8	79.4	55.1	54.1	55.6
Pit latrine	No	98.1	92.5	85.3	98.0	93.8
	Yes	1.9	7.5	14.7	2.1	6.3
Cattle ownership	No	17.7	55.7	89.8	81.3	58.4
	Yes	82.3	44.3	10.2	18.7	41.7
*Site characteristics*						
Baseline prevalence (TF and/or TI)*		81.0	68.0	52.0	74.0	70.0

TF, trachomatous inflammation-follicular; TI, trachomatous inflammation-intense

*Derived from the baseline survey [17]

### Prevalence of A,F,E interventions

Table 1 summarises the uptake of A,F,E interventions by study district. Overall, 53.0% of the eligible children had received at least one treatment with azithromycin, whereas 62.4% had a clean face on examination. Among the 706 households with eligible children: 72.5% reported washing faces of children two or more times a day; 73.1% had received health education; 44.4% had water accessible within 30 minutes; and only 6.3% had pit latrines.

### Ordinal logistic regression analysis of associations between severity of active trachoma signs and A,F,E interventions

Univariate ordinal logistic regression analysis of the associations between severity of active trachoma and A,F,E interventions is shown on Table 2. Factors associated with reduced odds of a more severe active trachoma sign compared with not having the risk factor were: older age (5–9 years compared to 1–4 years) [odds ratio (OR) = 0.3; 95% confidence interval (CI) (0.5–0.9)]; female sex (OR = 0.7; 95% CI 0.5–0.9); azithromycin treatment (one, two, or three treatments compared with no treatments) (p-trend<0.001); clean face (OR = 0.2; 95% CI 0.1–0.2); increased frequency of face washing (twice or more frequent daily face washing compared to once) (p-trend = 0.001); and pit latrine (OR = 0.4; 95% CI 0.2–0.9). There was no association between active trachoma severity and health education, and water access. Cattle ownership was associated with increased relative odds of a more severe active trachoma sign; however, this was not statistically significant: OR = 1.4; 95% CI (1.0–2.1); p = 0.066.

**Table 2:** Univariable ordinal logistic regression analysis of association between severity of active trachoma (no TF, no TI; TF only; any TI) and A,F,E interventions.

Factors	No. of Children (n = 1,7,12)	Prevalence (%)			Odds Ratio	95% CI	p-value
		No TF, no TI	TF Only	Any TI			
Age group (years)							
1–4	818	56%	27%	17%	1.0		
5–9	894	71%	19%	9%	0.3	0.2–0.4	<0.001
Sex							
Male	873	62%	24%	14%	1.0		
Female	839	67%	22%	11%	0.7	0.5–0.9	0.007
**A**ntibiotics							
Azithromycin treatment							
None	804	49%	29%	22%	1.0		p-trend <0.001
1 time	527	76%	18%	5%	0.8	0.5–1.0	
2 times	342	78%	18%	4%	0.5	0.3–0.8	
3 times	39	90%	8%	3%	0.1	0.0–0.4	
**F**acial cleanliness							
Clean face							
No	643	36%	40%	24%	1.0		
Yes	1069	81%	13%	6%	0.2	0.1–0.2	<0.001
Face washing per day							
1 time or none	470	62%	20%	17%	1.0		p-trend = 0.001
2 times	784	62%	24%	14%	0.7	0.4–1.0	
3 or more times	458	69%	24%	6%	0.5	0.3–0.7	
Health education							
No	460	56%	25%	19%	1.0		
Yes	1252	67%	22%	10%	1.2	0.8–1.8	0.348
**E**nvironmental improvement							
Water access							
≤30 minutes	760	62%	20%	18%	1.0		
>30 minutes	952	66%	26%	9%	1.1	0.9–1.4	0.178
Pit Latrine							
No	1605	63%	24%	13%	1.0		
Yes	107	82%	15%	3%	0.4	0.2–0.9	0.038
Cattle ownership							
No	999	79%	16%	5%	1.0		
Yes	713	44%	33%	24%	1.4	1.0–2.1	0.066

AFE = Antibiotics, Facial cleanliness, Environmental improvement

TF, trachomatous inflammation-follicular; TI, trachomatous inflammation-intense

Table 3 shows the multivariable associations between active trachoma signs and A,F,E interventions adjusting for age, sex and baseline prevalence. The number of individual treatments with azithromycin was associated with reduced relative odds of having less severe active trachoma (p-trend = 0.036). Having received one or two treatments was associated with a 20% reduction in the relative odds of active trachoma; however, this was not statistically significant. Receiving three treatments was independently and strongly associated with reduction on the relative odds of active trachoma: OR = 0.1; 95% CI (0.0–0.7). Clean face was also strongly associated with independent reduction in the relative odds of active trachoma: OR = 0.3; 95% CI (0.2–0.4). Reports of washing faces of children two or more times was independently associated with reduced relative odds of active trachoma (p-trend = 0.001); whereas households using pit latrines had 60% reduction in relative odds of active trachoma: OR = 0.4; 95% CI (0.2–0.9). There was no evidence of interaction between the A,F,E interventions based on our effect modification models. Sensitivity analysis of the effect of missing data by multiple imputation of missing data and analysis of association between severity of active trachoma and A,F,E interventions revealed effect estimates similar to those in which missing data had been excluded (data not shown).

**Table 3:** Multivariable ordinal logistic regression analysis of association between severity of active trachoma (no TF, no TI; TF only; any TI) and A,F,E interventions (n = 17,12).

Factor	Odds Ratio*	95% CI	p-value
**A**ntibiotics (azithromycin treatment)			
1 time	0.8	0.5–1.1	p-trend = 0.036
2 times	0.8	0.5–1.4	
3 times	0.1	0.0–0.7	
**F**acial cleanliness			
Clean face	0.3	0.2–0.4	<0.001
Face washing per day (twice)	0.7	0.4–1.0	p-trend = 0.001
Face washing per day (thrice or more)	0.4	0.3–0.7	
**E**nvironmental improvement			
Pit latrine	0.4	0.2–0.9	0.031

*Adjusted for the effects of age, sex and baseline prevalence

## Discussion

We have previously published findings on the effects of three years of the SAFE strategy in Southern Sudan based on a before-and-after analysis comparing change in prevalence of active trachoma signs at baseline compared to the three-year evaluation; which suggested that where more A,F,E activities had been implemented, greater declines in prevalence were observed [8]. In this study we present a cross-sectional analysis of associations between active trachoma and A,F,E components of the SAFE strategy. The cross-sectional nature of our data had limitations in that it did not take into account all the baseline risk factors that may influence the outcome. Modelling associations between change in prevalence of active trachoma and interventions accounting for change in risk factors would yield more precise results. However, this would not have been possible since the triennial evaluation was not designed to measure change in risk factors. Further research for a more rigorous method of modelling the effect of A,F,E interventions using cross-sectional survey data accounting for change in potential risk factors is suggested.

Despite the limitations in the study design, our findings suggest that receiving three treatments with azithromycin, clean face, increased frequency of washing faces of children and use of pit latrines were independently associated with reduced prevalence of active trachoma. The A,F,E interventions are designed to reduce the risk of trachomatous inflammation in individuals and communities; leading to a reduction in the lifetime risk of conjunctival scarring and blinding complications of trachoma. The effect estimates are consistent with independent effects of A,F,E interventions and reinforce the argument for implementing the full SAFE strategy as an integrated approach to control blinding trachoma. The packaging of trachoma control interventions into a four-pronged community-based approach provides a comprehensive programme for trachoma elimination that is adaptable to many different situations and which can be implemented at the community level. Because each component of the SAFE strategy uses appropriate and readily adaptable technologies, trachoma control can be integrated with broader health and development efforts targeting poor and marginalized populations [25].

Our survey was designed as an evaluation of the SAFE strategy under operational field conditions. We used clinical signs of active trachoma as operational outcome measures and maintained a high inter-observer reliability to minimize bias among trachoma graders, as recommended by the WHO [26]. It has been argued that nucleic acid amplification techniques (NAATs) of conjunctival swabs are the best way of determining the prevalence of ocular chlamydial infection in order to monitoring mass antibiotic administration [27]; however, this is currently not advocated by the WHO for programmatic use. We measured health education, face washing, and azithromycin treatment based on self-reports by the female heads of households and caregivers of children. Reporting of face washing behaviour by mothers or caregivers of children was likely to be overestimated since in most cultures hygiene is considered a desirable activity regardless of the actual practices [28]. However, recall bias in reporting of azithromycin treatment in children by caregivers was likely to underestimate azithromycin usage to a large extent. While the magnitude of these biases cannot be estimated, our study showed independent associations of antibiotics treatment, clean face, face washing and pit latrines and lower prevalence of active trachoma signs. Use of multilevel models controlled for clustering and variability of our observations at the household, village and district levels as well as the potential confounding effects of age, sex and baseline district prevalence of active trachoma.

We defined a clean face as the absence of ocular and or nasal discharge. Face washing has been shown to be effective in improving facial cleanliness in children [3]. Having a clean face is though to reduce autoinfection with ocular Chlamydia as well as reducing transmission to others. However, active trachoma also causes ocular and nasal discharge; therefore it is unclear whether dirty faces lead to trachoma, or trachoma results in dirty faces, especially in cross-sectional studies [29]. Consistent with previous studies [30], our models showed that a clean face was independently associated with lower prevalence of active trachoma. We also found face washing two or more times a day to be independently associated with lower prevalence of active trachoma. Nonetheless, there was no evidence of statistical interaction between face washing and cleans faces and between the A,F,E interventions, possibly because our sample was not powered to investigate such interactions. Overall, our study suggests that children who lived in families that were more amenable to receiving mass distribution of azithromycin, owned a pit latrine, and had faces washed two or more times a day, were less likely to have active trachoma.

Previous studies have explored the relationship between active trachoma and interventions based on ecological analysis [31]–[34]. However, ecological analyses have a major limitation since bias may result by generalisation of correlation of observations made at a group level to individuals. Our study has advantages since we have modelled individual outcomes of active trachoma. The ordinal category of active trachoma severity is more precise than the conventional dichotomous approach since it makes full use of the data on active trachoma signs that is inherent in the WHO simplified grading scheme. Variability in characteristics across the study districts has previously been highlighted as a possible explanation for heterogeneity in uptake of SAFE and therefore likely to affect the effectiveness of SAFE [8]. We used multilevel models which allowed for variability at individual, households and district levels as well as adjustment for non-independence of observations at household and district levels (clustering).

### Conclusions

This study of associations between the A,F,E components of SAFE and active trachoma showed independent protective effects against active trachoma of mass systemic azithromycin treatment, clean face on examination, reported face washing, and presence and use of pit latrines in the household. This study provides important evidence for continued advocacy for implementation of the full SAFE strategy for trachoma control.

**Box 1. The SAFE strategy for trachoma control**

- **S**urgery: eyelid surgery for the in-turned eyelashes to stop the pain and minimize risk of corneal damage.
- **A**ntibiotics: treatment for active trachoma using single-dose oral azithromycin (Zithromax) and tetracycline eye ointment.
- **F**acial cleanliness: clean faces especially in children through sustained hygiene behaviour change.
- **E**nvironmental improvement: to increase access to water and sanitation.
